# Integrated ADRC and Consensus Control for Anti-Disturbance Formation Tracking Control of Multiple Biomimetic Underwater Spherical Robots

**DOI:** 10.3390/biomimetics11040273

**Published:** 2026-04-15

**Authors:** Xihuan Hou, Miao Xu, Liang Wei, Hongfei Li, Zan Li, Huiming Xing, Shuxiang Guo

**Affiliations:** 1 School of Intelligence and Information Engineer, Tangshan University, Tangshan 063000, China; houxihuan@tsc.edu.cn (X.H.); weiliangts@163.com (L.W.); hongfeili@126.com (H.L.); 2 Cai Hong Unmanned Aerial Vehicle Co., Ltd., China Academy of Aerospace Aerodynamics, Beijing 100074, China; lizan@bit.edu.cn; 3 College of Intelligent Systems Science and Engineering, Harbin Engineering University, Harbin 150001, China; xinghuiming@hrbeu.edu.cn; 4 Department of Electronic and Electrical Engineering, Southern University of Science and Technology, Shenzhen 518055, China

**Keywords:** formation tracking control, active disturbance rejection control, consensus control, biomimetic underwater spherical robot

## Abstract

To facilitate the practical deployment and engineering implementation of multi-robot coordination for biomimetic underwater spherical robots (BUSRs), it is imperative to develop a formation tracking control method with a simple structure, a small number of tunable parameters, convenient parameter tuning and strong anti-disturbance capability. This study proposes a formation controller integrating virtual structure (VS), consensus protocol, and parallel output-velocity-type active disturbance rejection control (POV-ADRC), denoted as VS-C-POV-ADRC. A rotating global (RG) coordinate system is established to decouple robot positions from heading angles, which makes the parameter tuning more convenient. A double-loop control architecture is constructed, where the outer consensus control loop generates the desired velocity for each robot based on virtual-structure reference positions, and the inner POV-ADRC loop achieves high-precision velocity tracking. The proposed controller features a compact structure with only five adjustable parameters per motion direction, realizing easy engineering implementation and adaptation to the limited computing capacity of BUSRs. The simulation and experiment results demonstrate that the proposed algorithm enables robots to maintain a stable formation and achieve trajectory tracking accuracy within one body length, while exhibiting superior disturbance rejection. The proposed method provides a feasible and practical solution for BUSR formation control.

## 1. Introduction

In recent years, autonomous underwater vehicles (AUVs) have played an increasingly critical role in marine resource exploration and environmental monitoring [1]. However, traditional AUVs often lack the maneuverability required for operations in complex, cluttered, or narrow nearshore environments. To address these challenges, bio-inspired underwater robots have garnered significant attention due to their superior agility and adaptability [2,3]. Compared with a single bio-inspired underwater robot, a coordinated group of multiple underwater robots is more capable and flexible in performing complicated, large-scale and high-efficiency missions. Formation tracking control is a core issue associated with multi-robot systems, which has attracted growing attention and achieved significant progress.

Formation tracking control refers to controlling a multi-robot system to move along a desired trajectory while maintaining a specified formation. Various control frameworks have been designed for formation tracking problems. Generally, control frameworks consist of two layers: the upper layer is the formation strategy, and the lower layer is the control method. At present, three formation strategies are most widely adopted in relevant research: the leader–follower method [4,5,6], the behavior-based method [7,8], and the virtual structure scheme [9,10,11]. The leader–follower method is the simplest formation strategy and thus has been widely adopted. However, the excessive dependence on the leaders leads to poor reliability. In the behavior-based approach, the control motion is derived from a weighted average of the behaviors corresponding to each subtask [12]. The major drawback of this method lies in the absence of an explicit definition for the group behavior. The virtual-structure approach was proposed by Tan. This method regards the multi-robot system as a rigid body. Generally, there is a virtual leader as the global reference for motion. Actions of the other robots are derived according to the formation shape and the global reference motion. A core advantage of this method lies in its ability to strictly maintain the desired formation configuration while effectively avoiding error accumulation. In addition, this method decouples the complex multi-robot cooperative control problem into a trajectory tracking problem for a single robot. Features such as clear control logic, a simple implementation process and high system robustness make the method highly applicable to engineering practice.

To achieve formation control for multi-robot systems, the design of a reliable controller is critical. Currently, various control methods have been widely adopted for this task, including the backstepping method, model predictive control (MPC), adaptive control, consensus algorithms, and reinforcement learning (RL) algorithms. Fu et al. [13] developed a novel rigid graph-based model predictive controller (RGMPC) for formation control of AUVs. The proposed method avoided issues such as control jumps and thrust saturation while enhancing robustness. In addition, constraints were constructed using that method. In [14], a distributed nonlinear MPC scheme integrated with control barrier functions (CBFs) was proposed for trajectory tracking and formation control of multiple underwater robots.

In terms of backstepping-based methods, Yan et al. proposed a distributed robust-learning-based backstepping control method combined with neurodynamics, addressing engineering challenges such as completely unknown system parameters, model mismatches, and environmental disturbances for underwater vessels [15]. Additionally, a novel formation control law derived from the backstepping-sliding mode method was presented in [16], tailored for cooperative formation control of multiple AUVs under intermittent and unreliable underwater acoustic communication.

For adaptive control, Wan et al. proposed a distributed adaptive control scheme integrated with fuzzy logic approximation, which approximated complex nonlinear dynamics [17]. Although numerical simulations verified its effectiveness, the method involved a large number of tunable parameters. Regarding RL-based approaches, the paper [18] introduced a novel formation control and obstacle avoidance scheme based on deep reinforcement learning (DRL), but it only underwent simulation validation. Due to the complexity of the underwater environment, the well-trained simulation model exhibited poor adaptability to real-world conditions, making it difficult for practical deployment on physical robots. Consensus algorithms are also commonly utilized for underwater robot formation control. A consensus-based optimal coordination protocol was designed to solve the formation tracking problem of multiple AUVs in [19]. Wang et al. [6] presented a fixed-time event-triggered obstacle avoidance consensus control for a multi-AUV time-varying formation system in a 3D environment, incorporating an improved artificial potential field for obstacle avoidance.

Underwater robots face severe environmental disturbances in complex marine conditions, which greatly undermine the stability of the system and exert an adverse impact on its reliable operation [20]. To address this, sliding mode control (SMC) and active disturbance rejection control (ADRC) are widely adopted for their anti-disturbance capabilities. Wang et al. proposed an algorithm integrating SMC and disturbance observer (DO) to achieve effective anti-disturbance [21]. In [12], a robust formation tracking control scheme incorporating a distributed adaptive sliding-mode predictive controller was developed to overcome model uncertainties and external disturbances, with a rolling-time-horizon optimization technique integrated to improve convergence speed. Han et al. [22] proposed an ADRC technique that was capable of efficiently estimating various types of disturbances and had higher estimation accuracy. Ref. [23] proposed a distributed anti-disturbance formation control scheme integrating extended state observers (ESOs) and tracking differentiators (TD) to handle communication delay heterogeneity and strong nonlinear disturbances. Wang et al. presented an ADRC approach-based dynamic controller for a multi-AUV formation. The experiment results verified the effectiveness of the ADRC [24]. In [25], a distributed anti-disturbance control scheme combining a self-triggered mechanism and ADRC was proposed to handle the uncertain disturbance.

While all the aforementioned methods can achieve formation tracking, most rely on superimposing multiple simple controllers to ensure effectiveness and robustness. This practice inevitably results in complex controller structures and numerous tunable parameters, which restricts their practical application. In addition, most current studies remain at the stage of simulation verification. Bio-inspired underwater spherical robots feature compact structure, high flexibility, strong environmental adaptability, and low noise, making them particularly suitable for narrow nearshore waters. However, such robots also suffer from strong nonlinear dynamics, limited onboard computing resources, and high sensitivity to external environmental disturbances, which bring significant challenges to formation coordination. Therefore, it is urgent to develop a formation control scheme with a simple structure, few tunable parameters, strong anti-disturbance ability, and ease of engineering deployment. To address this critical gap, this paper proposes a formation tracking control method based on the virtual structure strategy and a dual-loop controller. Specifically, the virtual structure method is used to generate reference information (position and attitude) for each individual robot. The dual-loop controller consists of a consensus algorithm (outer loop) and ADRC (inner loop): the outer loop leverages the consensus algorithm to generate the desired velocity for each robot, ensuring formation consistency; the inner loop utilizes ADRC to track the desired velocity, achieving high-precision trajectory tracking with strong anti-disturbance performance. The main contributions of this article are as follows:

(1) In order to simplify the design of the formation tracking controller, a global rotating frame is proposed, which splits the position information and heading angle.
(2) A dual-loop control architecture integrating consensus and ADRC is proposed to address the inherent limitations of single-loop control. Directly decomposing the formation tracking task into individual trajectory tracking and using only ADRC leads to formation instability due to the lack of inter-robot communication. Although consensus algorithms are suitable for multi-robot formation, they typically rely on integral chain systems. Due to their strong nonlinear terms and external disturbances, bio-inspired underwater spherical robots are not integral chain systems. By combining consensus (outer loop for consistency) and ADRC (inner loop for anti-disturbance tracking), the proposed dual-loop architecture effectively resolves these contradictions.
(3) The proposed controller exhibits significant engineering viability. By requiring only five tunable parameters per motion direction, it alleviates the computational burden and simplifies deployment on platforms with constrained processing power.

The remainder of this paper is structured as follows: Section 2 formulates the modeling of the BUSR model. Design of the formation tracking method is described in Section 3. Simulation analysis and experimental verification are introduced in Section 4 and Section 5. Section 6 details the discussion. Finally, the conclusions and future work are summarized in Section 7.

## 2. Modeling of the Biomimetic Underwater Spherical Robot

### 2.1. Overview of the Biomimetic Underwater Spherical Robot

**Figure 1:**
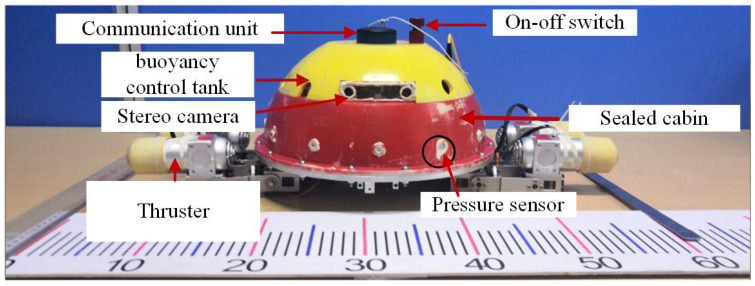
Prototype of the BUSR.

**Figure 2:**
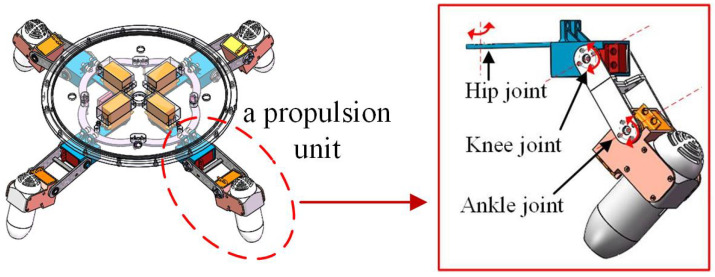
Vector propulsion system of the BUSR.

A biomimetic underwater spherical robot (BUSR) was designed, inspired by sea turtles, in our previous work [1,26]. The prototype is shown in Figure 1. The BUSR is divided into an upper and a lower section. The upper section contains a sealed cabin and a buoyancy control tank, while the lower section consists of a vector propulsion system. The vector propulsion system consists of four propulsion units, as shown in Figure 2. To adapt to the operational requirements in the narrow spaces of nearshore waters, the BUSR adopts a miniaturized design. Constrained by the volume, the robot is equipped with a limited number of onboard sensors and modest hardware computing capabilities, which poses significant challenges to the design of the controller. However, the spatial arrangement of the four propulsion units is adjusted flexibly according to actual operational demands, which endows the robot with excellent locomotion flexibility. Some key technical parameters are listed in Table 1.

### 2.2. Description of the BUSR Model

**Figure 3:**
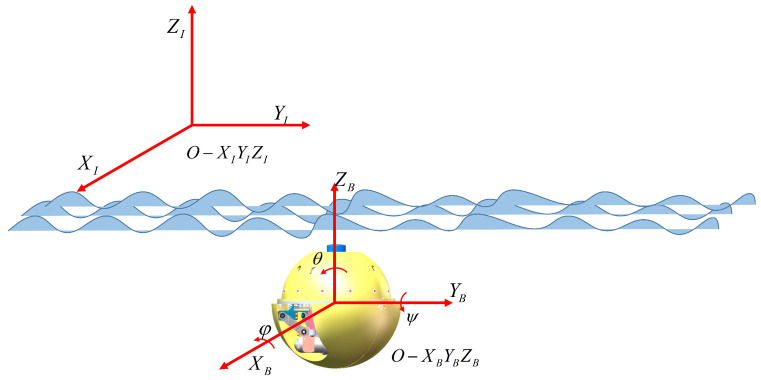
Definition of the inertial frame and body frame.

In order to describe the dynamic and kinematic model of the robot, two frames are built as shown in Figure 3.

The posture of the robot in the $O-X_{I}Y_{I}Z_{I}$ is denoted as follows:

(1) $$\eta ={\left[x,y,z,\phi ,\psi ,\theta \right]}^{T}$$

where *x*, *y*, and *z* represent the position. ϕ, ψ, and θ denote the roll, pitch and yaw angles about the $X_{I}$, $Y_{I}$, and $Z_{I}$ axes. Velocities of the robot are denoted as follows in the $O-X_{B}Y_{B}Z_{B}$ frame:

(2) $$v={\left[u,\nu ,w,p,q,r\right]}^{T}$$

where *u*, ν, and *w* represent the surge, sway, and heave velocities along the $X_{B}$, $Y_{B}$, and $Z_{B}$ axes, respectively. *p*, *q*, and *r* are the roll, pitch and yaw angular velocities rotating about the $X_{B}$, $Y_{B}$, and $Z_{B}$ axes, respectively. The kinematic model of the robot can be expressed as follows:

(3) $$\dot{\eta }=Jv$$

Considering that six degrees of freedom (DOFs) are not all subject to control when the robot performs 3D underwater motion, it is sufficient to control the variables *x*, *y*, *z*, and θ to enable the robot to reach any arbitrary point underwater. Therefore, the degrees of roll and pitch are neglected in this paper. Let $\eta ={\left[x,y,z,\theta \right]}^{T}$ and $v={\left[u,\nu ,w,r\right]}^{T}$. J in Equation (3) is written as follows:

(4) $$J=\left[\begin{array}{cccc} c\theta & -s\theta & 0 & 0\\ s\theta & c\theta & 0 & 0\\ 0 & 0 & 1 & 0\\ 0 & 0 & 0 & 1 \end{array}\right]$$

where cθ is cosθ and sθ is sinθ. In addition, the BUSR has the following characteristics: (1) The robot operates at a low speed, typically below 0.5 m/s. (2) The effect of the rotation of the Earth on the motion of the robot is negligible. (3) The underwater robot adopts a compact structure with coincident center of gravity and buoyancy. During low-speed cruising, the roll and pitch angles are maintained within a very small range (less than ±5° in experiments), which has a negligible influence on the position and heading control. Therefore, the dynamic model of the BUSR is simply written as follows:

(5) $$M\dot{v}+D\left(v\right)v=\tau$$

where $\tau ={\left[\tau_{u},\tau_{\nu },\tau_{w},\tau_{r}\right]}^{T}$. $M\in R^{4\times 4}$ is denoted as:

(6) $$\left[\begin{array}{cccc} m_{11} & 0 & 0 & 0\\ 0 & m_{22} & 0 & 0\\ 0 & 0 & m_{33} & 0\\ 0 & 0 & 0 & m_{44} \end{array}\right]$$

${D(v)\in R}^{4\times 4}$ is denoted as:

(7) $$\left[\begin{array}{cccc} X_{u} & 0 & 0 & 0\\ 0 & Y_{v} & 0 & 0\\ 0 & 0 & Z_{w} & 0\\ 0 & 0 & 0 & N_{r} \end{array}\right]+\left[\begin{array}{cccc} X_{u|u|}\left|u\right| & 0 & 0 & 0\\ 0 & Y_{v|v|}\left|v\right| & 0 & 0\\ 0 & 0 & Z_{w|w|}\left|w\right| & 0\\ 0 & 0 & 0 & N_{r|r|}\left|r\right| \end{array}\right]$$

Accordingly, the simplified mathematical model of the robot can be formulated as follows:

(8) $$\left\{\begin{array}{c} \dot{\eta }=J\left(\theta \right)v\\ \dot{v}=M^{-1}\tau -M^{-1}D\left(v\right)v \end{array}\right.$$

where the second equation in Equation (8) is expanded as follows:

(9) $$\left\{\begin{array}{c} \dot{u}=a_{11}u+a_{12}u^{2}+b_{1}\tau_{u}\\ \dot{\nu }=a_{21}\nu +a_{22}\nu^{2}+b_{2}\tau_{\nu }\\ \dot{w}=a_{31}w+a_{32}w^{2}+b_{3}\tau_{w}\\ \dot{r}=a_{41}r+a_{42}r^{2}+b_{4}\tau_{r} \end{array}\right.$$

Parameters in the above model are obtained based on the least squares method using the experimental data of the underwater motion. The specific values of the hydrodynamic parameters are given in Table 2. In practice, the values of the hydrodynamic parameters fluctuate slightly with the different operating states of the robot.

### 2.3. Thrust Allocation Method of the X-Shaped Motion Mode

**Figure 4:**
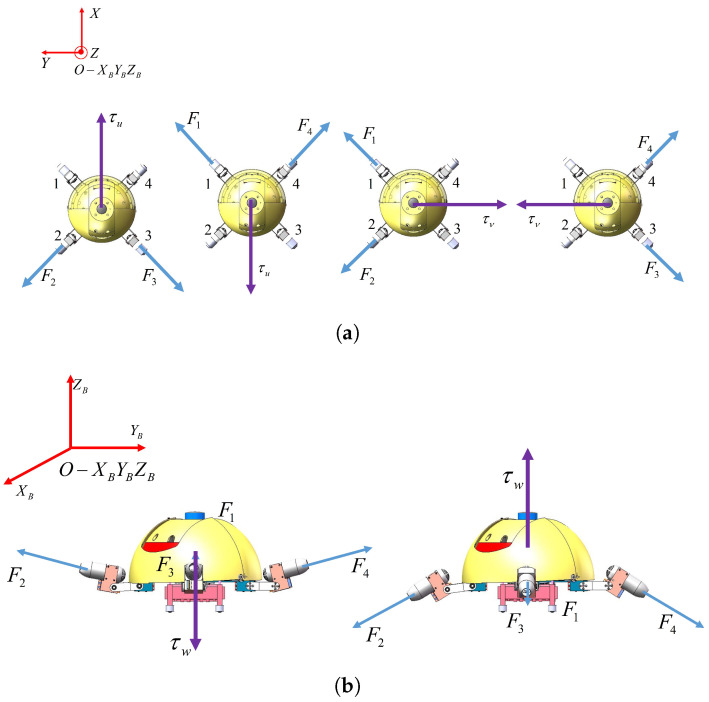
X-shaped motion mode. (**a**) X-shaped motion mode in the top view. (**b**) X-shaped motion mode in the side view.

The X-shaped motion mode refers to a locomotion pattern where the front two legs of the robot are positioned at a 45° angle to the oblique front, while the rear two legs are oriented at a 45° angle to the oblique rear, as illustrated in Figure 4. The angle between adjacent legs is 90°, and the four drive units of the robot show an “X” shape. In this mode, the robot achieves three degrees of freedom (DOFs) of motion, namely surge, sway, and heave motion. The resultant force/moment vector of the robot is synthesized according to Equation (10):

(10) $${\tau =B}_{X}F$$

where the vector $F={\left[F_{1},F_{2},F_{3},F_{4}\right]}^{T}$ describes the thrust magnitudes of the four thrusters. Matrix $B_{X}\in R^{4\times 4}$ is specified below:

(11) $$B_{X}=\left[\begin{array}{cccc} -c\chi_{1}/\sqrt{2} & c\chi_{2}/\sqrt{2} & c\chi_{3}/\sqrt{2} & -c\chi_{4}/\sqrt{2}\\ -c\chi_{1}/\sqrt{2} & -c\chi_{2}/\sqrt{2} & c\chi_{3}/\sqrt{2} & c\chi_{4}/\sqrt{2}\\ -s\chi_{1} & -s\chi_{2} & -s\chi_{3} & -s\chi_{4}\\ 0 & 0 & 0 & 0 \end{array}\right]$$

where $\chi_{i}$(i=1,2,3,4) represents the angles between the thrust directions and the horizontal direction. Considering the mechanical structure design, the limited range of servo angles, and the center-of-gravity balance during robot motion, $\chi_{i}$ is constrained to $-\pi /6\leq \chi_{i}\leq \pi /6$.

**Figure 5:**
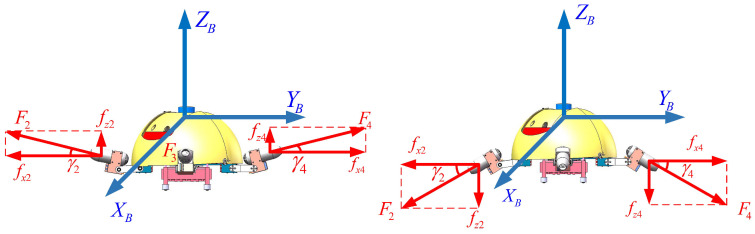
Thrust calculation in X-shaped motion mode.

Thrust calculation for the thrusters is represented in Figure 5. The thrust of the *i*th thruster can be decomposed into a horizontal component $f_{xi}$ and a vertical component $f_{zi}$. The robot is driven by the thrust differences between the thrusters. Thus, we first set a horizontal thrust reference value and a vertical thrust reference value. The equation for obtaining the offset values is as follows:

(12) $$\left\{\begin{array}{c} \Delta f_{ux2}+\Delta f_{ux3}-\left(\Delta f_{ux1}+\Delta f_{ux4}\right)=\sqrt{2}\tau_{u}\\ \Delta f_{\nu x3}+\Delta f_{\nu x4}-\left(\Delta f_{\nu x1}+\Delta f_{\nu x2}\right)=\sqrt{2}\tau_{\nu }\\ \Delta f_{wz1}+\Delta f_{wz2}+\Delta f_{wz3}+\Delta f_{wz4}=\tau_{w} \end{array}\right.$$

where $\Delta f_{uxi}$(i=1,2,3,4), $\Delta f_{\nu xi}$(i=1,2,3,4) and $\Delta f_{wxi}$(i=1,2,3,4) represent the thrust offset to generate surge, sway, and heave motion, respectively. For the convenience of calculation, let the absolute offset values of the four thrusts $\Delta f_{uxi}$ be equal. According to Equation (12), $\Delta f_{ux2}$ and $\Delta f_{ux3}$ are equal to $\sqrt{2}\tau_{u}/4$, and $\Delta f_{ux1}$ and $\Delta f_{ux4}$ are equal to $-\sqrt{2}\tau_{u}/4$. The values of $\Delta f_{\nu xi}$ are determined similarly. The thrust offsets of heave motion $\Delta f_{wz1}$, $\Delta f_{wz2}$, $\Delta f_{wz3}$ and $\Delta f_{wz4}$ are all equal to $\tau_{w}/4$. Consequently, the thrusts of the four thrusters in the horizontal and vertical directions are obtained, as shown in Equations (13) and (14).

(13) $$\left\{\begin{array}{c} f_{x1}=f_{x-base}+\left(-\sqrt{2}\tau_{u}/4\right)+\left(-\sqrt{2}\tau_{\nu }/4\right)\\ f_{x2}=f_{x-base}+\sqrt{2}\tau_{u}/4+\left(-\sqrt{2}\tau_{\nu }/4\right)\\ f_{x3}=f_{x-base}+\sqrt{2}\tau_{u}/4+\sqrt{2}\tau_{\nu }/4\\ f_{x4}=f_{x-base}+\left(-\sqrt{2}\tau_{u}/4\right)+\sqrt{2}\tau_{\nu }/4 \end{array}\right.$$

(14) $$\left\{\begin{array}{c} f_{z1}=f_{z-base}+\tau_{w}/4\\ f_{z2}=f_{z-base}+\tau_{w}/4\\ f_{z3}=f_{z-base}+\tau_{w}/4\\ f_{z4}=f_{z-base}+\tau_{w}/4 \end{array}\right.$$

The thrust of each thruster can be obtained according to Equation (15). The ankle joint angle is given by Equation (16).

(15) $$F_{i}=\sqrt{f_{xi}^{2}+f_{zi}^{2}}$$

(16) $$\chi_{i}=\arctan\left(\frac{f_{zi}}{f_{xi}}\right)$$

## 3. Design of Formation Tracking Method

**Figure 6:**
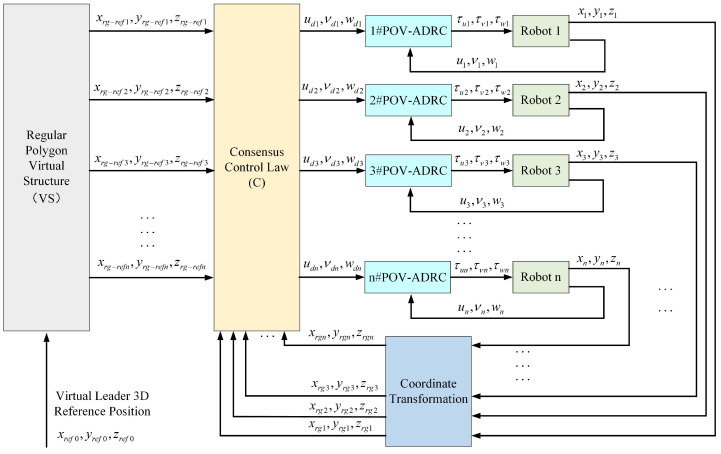
VS-C-POV-ADRC formation control framework.

In order to achieve formation tracking control for multiple BUSRs in environments with significant disturbances, this paper designed a formation algorithm that combines the double-loop formation tracking controller based on consensus-ADRC with the virtual structure approach, as shown in Figure 6. The formation algorithm consists of the following three steps. First, the desired position of each robot in the formation system is obtained according to the global formation reference trajectory based on the virtual structure. Subsequently, the desired velocity is derived based on the outer-loop consensus controller. Finally, parallel output-velocity-type ADRC is designed to track the desired velocity. Thereafter, each step in the formation algorithm is introduced in detail.

### 3.1. Building of a Rotating Global Frame

**Figure 7:**
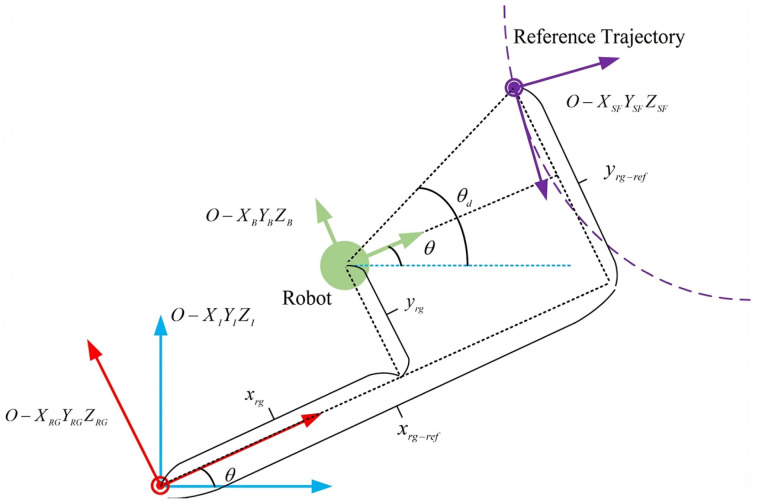
Relationship among different coordinate systems.

To facilitate the design of the ADRC controller, a rotating global (RG) coordinate system was designed, denoted as $O-X_{RG}Y_{RG}Z_{RG}$ (named RG coordinate system), as shown in Figure 7.

The origin of the RG coordinate system coincides with that of the inertial coordinate system $O-X_{I}Y_{I}Z_{I}$, while the orientation is continuously aligned with that of the robot. In other words, the axes of the RG frame are parallel to the respective axes of the body frame of the robot $O-X_{B}Y_{B}Z_{B}$, differing only in the location of their origins.

The fixed inertial coordinate system easily leads to trigonometric nonlinear terms in the Linear Extended State Observer (LESO) of the ADRC. This causes the observer bandwidth to vary continuously, which may adversely affect disturbance observation. In contrast, the RG coordinate system is able to fix the LESO bandwidth, which ensures a stable estimation of disturbance. Furthermore, the RG coordinate system is able to isolate the position from the heading angle, facilitating the parameter tuning of the ADRC. Since the RG coordinate system rotates synchronously with the robot, the attitude angles of the robot ϕ, ψ, and θ in the RG coordinate system are all zero. The transformation of the position of the robot from the inertial frame $O-X_{I}Y_{I}Z_{I}$ to the RG frame $O-X_{RG}Y_{RG}Z_{RG}$ can be expressed as follows:

(17) $$\left[\begin{array}{c} x_{rg}\\ y_{rg}\\ z_{rg} \end{array}\right]=\left[\begin{array}{ccc} c\theta & s\theta & 0\\ -s\theta & c\theta & 0\\ 0 & 0 & 1 \end{array}\right]\left[\begin{array}{c} x\\ y\\ z \end{array}\right]$$

Furthermore, by combining the kinematic and dynamic models, the dynamic model of the robot in the $O-X_{rg}Y_{rg}Z_{rg}$ coordinate system can be obtained as:

(18) $$\left\{\begin{array}{c} {\ddot{x}}_{rg}=a_{11}{\dot{x}}_{rg}+a_{12}{\dot{x}}_{rg}^{2}+b_{1}\tau_{u}\\ {\ddot{y}}_{rg}=a_{21}{\dot{y}}_{rg}+a_{22}{\dot{y}}_{rg}^{2}+b_{2}\tau_{\nu }\\ {\ddot{z}}_{rg}=a_{31}{\dot{z}}_{rg}+a_{32}{\dot{z}}_{rg}^{2}+b_{3}\tau_{w}\\ \ddot{\theta }=a_{41}\dot{\theta }+a_{42}{\dot{\theta }}^{2}+b_{4}\tau_{r} \end{array}\right.$$

The first three equations are expressed in the RG coordinate system, and the fourth equation is in the inertial coordinate system.

Considering that disturbances caused by currents in the ocean are exerted on the robot in the form of forces, the mathematical model of the robot with current disturbance forces can be expressed as follows:

(19) $$\left\{\begin{array}{c} {\ddot{x}}_{rg}=a_{11}{\dot{x}}_{rg}+a_{12}{\dot{x}}_{rg}^{2}+b_{1}\tau_{u}+b_{1}\Omega_{fu}\\ {\ddot{y}}_{rg}=a_{21}{\dot{y}}_{rg}+a_{22}{\dot{y}}_{rg}^{2}+b_{2}\tau_{\nu }+b_{2}\Omega_{f\nu }\\ {\ddot{z}}_{rg}=a_{31}{\dot{z}}_{rg}+a_{32}{\dot{z}}_{rg}^{2}+b_{3}\tau_{w}+b_{3}\Omega_{fw}\\ \ddot{\theta }=a_{41}\dot{\theta }+a_{42}{\dot{\theta }}^{2}+b_{4}\tau_{r}+b_{4}\Omega_{fr} \end{array}\right.$$

In Equation (19), the first three equations are defined in the RG coordinate system, while the last equation is in the inertial coordinate system. $\Omega_{fu}$, $\Omega_{f\nu }$, $\Omega_{fw}$, and $\Omega_{fr}$ represent the current disturbance forces/torques acting on the robot in the surge, sway, heave, and yaw directions, respectively.

### 3.2. Modeling of the Regular Polygon Virtual Structure

**Figure 8:**
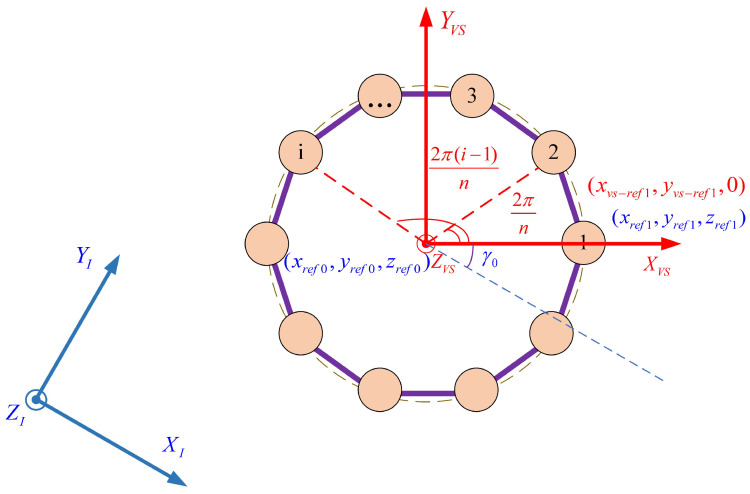
Regular Polygon Virtual Structure.

The virtual structure method treats the formation system as a rigid body. The vertex coordinates of the rigid polygon structure are assigned as the target positions for the individual robots in the formation system. In this paper, each small spherical robot is seen as a vertex of the virtual structure, and the center of regular polygon of the virtual structure is seen as a virtual leader. Without loss of generality, a regular polygon structure is established as shown in Figure 8. The circumradius of the polygon is set to $R_{vs}$. The parametric equation of the circumcircle in the virtual structure coordinate system $O-X_{VS}Y_{VS}Z_{VS}$ is given as follows.

(20) $$\left\{\begin{array}{c} x_{vs}=R_{vs}\cos\left(\theta_{vs}\right)\\ y_{vs}=R_{vs}\sin\left(\theta_{vs}\right)\\ z_{vs}=0 \end{array}\right.$$

If the regular polygon has *n* sides, then the reference position for the *i*th robot (i=1,2,…,n) in the $O-X_{VS}Y_{VS}Z_{VS}$ system, which corresponds to the vertex position, is given by Equation (21). Assume that the yaw angle of the virtual structure with respect to the inertial frame is $\gamma_{0}$. The reference position of the circumcenter in $O-X_{I}Y_{I}Z_{I}$ is ${\left[x_{ref0},y_{ref0},z_{ref0}\right]}^{T}$. The coordinate transformation for the reference position of the *i*th robot from the virtual system $O-X_{VS}Y_{VS}Z_{VS}$ to the inertial system $O-X_{I}Y_{I}Z_{I}$ is given by Equation (22):

(21) $$\left\{\begin{array}{c} x_{vs-refi}=R_{vs}\cos\left(2\pi \left(i-1\right)/n\right)\\ y_{vs-refi}=R_{vs}\sin\left(2\pi \left(i-1\right)/n\right)\\ z_{vs-refi}=0 \end{array}\right.$$

(22) $$\left[\begin{array}{c} x_{refi}\\ y_{refi}\\ z_{refi} \end{array}\right]=\left[\begin{array}{ccc} c\gamma_{0} & -s\gamma_{0} & 0\\ s\gamma_{0} & c\gamma_{0} & 0\\ 0 & 0 & 1 \end{array}\right]\left[\begin{array}{c} x_{vs-refi}\\ y_{vs-refi}\\ z_{vs-refi} \end{array}\right]+\left[\begin{array}{c} x_{ref0}\\ y_{ref0}\\ z_{ref0} \end{array}\right]$$

Furthermore, the reference positions of each robot in the RG frame can be obtained based on Equation (17), denoted as ${\left[x_{rg-refi},y_{rg-refi},z_{rg-refi}\right]}^{T}$. Subsequently, the formation tracking task can be accomplished by designing an effective controller to ensure that the robot accurately tracks the desired position.

### 3.3. Dual-Loop Formation Tracking Control Law Based on Consensus-ADRC

Directly applying Active Disturbance Rejection Control (ADRC) to each robot for formation tracking lacks robustness due to the absence of communication among robots. Conversely, traditional consensus protocols are often designed for integrator-chain systems to achieve formation. However, there are nonlinear terms and external disturbances in the dynamic model of the BUSR, which have a significant impact on the system. It is obvious that the consensus algorithm is not feasible for formations of multiple small spherical robots. Therefore, this paper proposes a dual-loop control law that integrates consensus control with ADRC. As illustrated in Figure 6, this controller operates as a dual-loop system.

The outer loop is a consensus-based controller. The current positions and reference positions of the robots are input signals. Based on the consensus protocol, the desired velocity for each robot is output.

The inner loop is an ADRC-based velocity tracker. The desired velocity provided by the outer loop is set as the reference signal. The output of the inner loop is the real control value that is exerted on the robots. The synergy of these two control laws achieves robust and accurate formation trajectory tracking.

#### 3.3.1. Communication Topology of the Formation System

Consider a formation system consisting of *n* small spherical robots, indexed by 1,2,…,n. The index of the virtual leader in the formation system is n+1. $G_{n+1}=\left(V,E\right)$ is a directed graph of the communication, which contains n+1 nodes and *n* edges. $V_{n+1}=\left\{1,2,3,\ldots ,n+1\right\}$ represents the set of nodes. $E_{n+1}\subseteq V_{n+1}\times V_{n+1}$ is the set of edges. $A_{n+1}=\left[a_{ij}\right]\in R^{\left(n+1)\times (n+1\right)}$ is the adjacency matrix. The elements of the adjacency matrix indicate whether there is a communication link between two robots. Specifically, for i∈{1,…,n} and j∈{1,…,n+1}, if there is a directed edge from node *j* to *i*, $a_{ij}=1$, otherwise $a_{ij}=0$. $L_{n+1}=\left[l_{ij}\right]\in R^{\left(n+1)\times (n+1\right)}$ is the Laplacian matrix associated with the graph $G_{n+1}$, where $l_{ij}=-a_{ij}$(i≠j), and $l_{ii}=\sum_{j=1,j\neq i}^{n+1}a_{ij}$(i,j=1,2,3,…,n+1).

#### 3.3.2. Consensus-Based Outer-Loop Control Algorithm

Considering that the control of actual spherical robots and the information interaction among robots are realized through switches and optical fibers, the following assumptions are supplemented in combination with the actual situation of multi-robot formation.

**Assumption** **1.** *Almost all robots in the formation system can receive the position of the virtual leader in real time.*

According to the above assumption, the outer-loop consensus controller for the *i*th robot is designed as follows:

(23) $$\left\{\begin{array}{c} \begin{array}{cc} u_{rg-di} & ={\dot{x}}_{rg-refi}+k_{1u}\{-\alpha_{i}\left[x_{rgi}-x_{rg-refi}\right]\\ \; & -\sum_{j=1}^{n}a_{ij}\left[\left(x_{rgi}-x_{rgj}\right)-\left(x_{rg-refi}-x_{rg-refj}\right)\right]\} \end{array}\\ \begin{array}{cc} \nu_{rg-di} & ={\dot{y}}_{rg-refi}+k_{1\nu }\{-\alpha_{i}\left[y_{rgi}-y_{rg-refi}\right]\\ \; & -\sum_{j=1}^{n}a_{ij}\left[\left(y_{rgi}-y_{rgj}\right)-\left(y_{rg-refi}-y_{rg-refj}\right)\right]\} \end{array}\\ \begin{array}{cc} w_{rg-di} & ={\dot{z}}_{rg-refi}+k_{1w}\{-\alpha_{i}\left[z_{rgi}-z_{rg-refi}\right]\\ \; & -\sum_{j=1}^{n}a_{ij}\left[\left(z_{rgi}-z_{rgj}\right)-\left(z_{rg-di}-z_{rg-refj}\right)\right]\} \end{array} \end{array}\right.$$

where $u_{rg-di}$, $\nu_{rg-di}$, and $w_{rg-di}$ represent the surge, sway and heave velocities of the *i*th robot, respectively. $x_{rg-refi}$, $y_{rg-refi}$, and $z_{rg-refi}$ represent the reference position information of the *i*th robot in the RG frame, which can be obtained based on the virtual structure.

#### 3.3.3. ADRC-Based Inner-Loop Control Algorithm

The outer-loop controller provides the desired velocity for the inner-loop controller. The dynamic model with disturbance of the robot is as follows:

(24) $$\left\{\begin{array}{c} {\dot{u}}_{rg}=a_{11}u_{rg}+a_{12}u_{rg}^{2}+b_{1}\tau_{u}+b_{1}\Omega_{fu}\\ {\dot{\nu }}_{rg}=a_{21}\nu_{rg}+a_{22}\nu_{rg}^{2}+b_{2}\tau_{\nu }+b_{2}\Omega_{f\nu }\\ {\dot{w}}_{rg}=a_{31}w_{rg}+a_{32}w_{rg}^{2}+b_{3}\tau_{w}+b_{3}\Omega_{fw} \end{array}\right.$$

To track the desired velocity provided by the outer-loop controller based on the X-shaped motion mode, ADRC must be applied individually to the three basic motions including surge, sway and heave. Based on the mathematical model presented in Equation (24), it is evident that the models for the surge, sway, and heave directions are formally identical and they are first-order nonlinear models. Therefore, a controller based on three parallel first-order ADRCs, named POV-ADRC, was designed. Given the similarity of the controllers across these three directions, only the design of the surge-direction ADRC controller is described in detail.

(1) Design of the Tracking-Differentiator (TD)

The primary role of the TD is to overcome noise disturbance and extract the input signal. The TD was designed according to the fastest comprehensive function (fhan) derived by Professor Han Jingqing:

(25) $$\left\{\begin{array}{c} f_{utrans}=-\left(u_{trans1}-u_{rg-ref}\right)\vartheta_{u}\\ u_{trans1}=u_{trans1}+hf_{utrans} \end{array}\right.$$

where $\vartheta_{u}$ is a speed factor that influences the tracking speed. *h* is the integral step size. $u_{trans1}$ is used to track $u_{rg-ref}$.

(2) Design of the LESO

Since the dynamic model of the robot is first-order, a second-order linear extended state observer (LESO) was designed to observe all uncertain terms.

(26) $$\left\{\begin{array}{c} {\tilde{u}}_{1}=u_{rg}-{\hat{u}}_{1}\\ {\hat{u}}_{1}={\hat{u}}_{1}+h\left({\hat{u}}_{2}+\lambda_{u1}{\tilde{u}}_{1}+b_{u}\tau_{u}\right)\\ {\hat{u}}_{2}={\hat{u}}_{2}+h\lambda_{u2}{\tilde{u}}_{1} \end{array}\right.$$

where ${\hat{u}}_{1}$ is the observation value of $u_{rg}$. ${\hat{u}}_{2}$ represents the observed value of all uncertain terms. To facilitate parameter adjustment and minimize the number of parameters in the controller, $\lambda_{u1}$ and $\lambda_{u2}$ in the above equation were set to $2\omega_{u}$, $\omega_{u}^{2}$ respectively. For the second-order LESO mentioned above, only one parameter, the bandwidth $\omega_{u}$, needs to be adjusted. When the system sampling frequency is sufficiently high, a larger bandwidth needs to be set to accurately observe the uncertain terms of the system.

(3) Design of the LSEF

The linear state error feedback (LSEF) controller primarily counteracts disturbances and eliminates the output error of the TD and ESO. The state error feedback controller essentially functions as a proportional–derivative (PD) controller for the ADRC. The error compensation controller is designed as follows:

(27) $$\left\{\begin{array}{c} u_{e1}=u_{trans1}-{\hat{u}}_{1}\\ \tau_{u}=f_{utrans}+\beta_{u}u_{e1} \end{array}\right.$$

where $\tau_{u0}$ is the output of the LSEF. The parameter $\beta_{u0}$ is a parameter that needs to be adjusted. However, the actual control value shown in Equation (27) needs to include the observed disturbance.

(28) $$\tau_{u}=\frac{\tau_{u}-{\hat{u}}_{2}}{b_{u}}$$

The above controller contains 4 parameters: $\vartheta_{u}$, $\omega_{u}$, $\beta_{u}$, and $b_{u}$. To ensure the observation accuracy of uncertain terms including disturbance, the bandwidth of the LESO can be set relatively large. When the parameter $b_{u}$ is adjusted to approach $b_{1}$, the disturbance observation term accurately observes the nonlinear terms of the system.

### 3.4. Stability Analysis

Since the yaw angle adjustment is unavailable for the robot in the X-shaped motion mode, when the robot tracks the desired trajectory, the heading is not required to be consistent with the reference trajectory. In addition, based on the characteristics of the spherical robot operating in water, the following assumptions are made:

**Assumption** **2.** *The input–output relationship of the actuators in the motion control system is approximately 1.*

According to the above designed formation controller, the following theorem is given:

**Theorem** **1.** *If the above assumption is satisfied, to successfully complete the trajectory tracking task based on VS-C-POV-ADRC, the following conditions must be satisfied: $\vartheta_{ui}>0$, $\vartheta_{vi}>0$, $\vartheta_{wi}>0$ (i=1,2,…,n); $\omega_{u}>0$, $\omega_{v}>0$, and $\omega_{w}>0$ (i=1,2,…,n). The parameters must be set such that $b_{ui}\approx b_{1i}$, $b_{vi}\approx b_{2i}$, $b_{wi}\approx b_{3i}$ (i=1,2,…,n); $k_{1u}>0$, $k_{1v}>0$, $k_{1w}>0$; $\beta_{ui}>0$, $\beta_{vi}>0$, $\beta_{wi}>0$, and $\alpha_{i}>0$ (i=1,2,…,n).*

**Proof** **of** **Theorem** **1.** Considering that the three degrees of freedom of the X-shaped motion mode are symmetrical, the proof can first be carried out for the surge degree of freedom. The model of the *i*th robot along the $x_{rg}$ direction is depicted as follows:

(29) $$\left\{\begin{array}{c} {\dot{x}}_{rgi}=u_{rgi}\\ {\dot{u}}_{rgi}=a_{11i}u_{rgi}+a_{12i}u_{rgi}^{2}+b_{1i}\tau_{ui}+b_{1i}\Omega_{fui} \end{array}\right.$$

The position $x_{rgi}$ is controlled via a cascaded approach. Because $u_{rgi}$ is not directly actuated, the control process begins with manipulating the input $\tau_{ui}$ to regulate the velocity $u_{rgi}$, which ultimately governs the position $x_{rgi}$. Firstly, we need to prove that $u_{rg-refi}$ is able to drive $x_{rgi}$ to converge to $x_{rg-refi}$. By substituting Equation (30) into ${\dot{x}}_{rgi}=u_{rgi}$, we obtain Equation (31).

(30) $$\begin{array}{cc} u_{rg-di} & ={\dot{x}}_{rg-refi}+k_{1u}\{-\alpha_{i}\left[x_{rgi}-x_{rg-refi}\right]\\ \; & -\sum_{j=1}^{n}a_{ij}\left[\left(x_{rgi}-x_{rgj}\right)-\left(x_{rg-refi}-x_{rg-refj}\right)\right]\} \end{array}$$

(31) $$\begin{array}{cc} {\dot{x}}_{rg-refi}-{\dot{x}}_{rgi} & =k_{1u}\{-\alpha_{i}\left(x_{rg-refi}-x_{rgi}\right)\\ \; & -\sum_{j=1}^{n}a_{ij}\left[\left(x_{rg-refi}-x_{rgi}\right)-\left(x_{rg-refj}-x_{rgj}\right)\right]\} \end{array}$$

Define ${\tilde{x}}_{rgi}=x_{rg-refi}-x_{rgi}$; then, the following equation is obtained:

(32) $${\dot{\tilde{x}}}_{rgi}=k_{1u}\left[-\alpha_{i}{\tilde{x}}_{rgi}-\sum_{i=1}^{n}a_{ij}\left({\tilde{x}}_{rgi}-{\tilde{x}}_{rgj}\right)\right]$$

The matrix form of the above equation is written as follows:

(33) $${\dot{\tilde{x}}}_{rg}=-k_{1u}\left(L_{n}+Q_{n}\right)x_{rg}$$

where $Q_{n}=diag\left\{\alpha_{1},\alpha_{2}\ldots ,\alpha_{n}\right\}$, $\alpha_{i}>0,\left(i=1,2,3,\ldots ,n\right)$. $L_{n}$ is the Laplacian matrix. $k_{1u}>0$. According to Theorem 3.6 in reference [27], it can be easily seen that the eigenvalues of $-k_{1u}\left(L_{n}+Q_{n}\right)$ have negative real parts. Therefore, ${\tilde{x}}_{rgi}$ is able to converge to zero. In other words, $x_{rgi}$ is able to converge to $x_{rg-refi}$. Secondly, we need to prove that $\tau_{ui}$ is able to drive $u_{rgi}$ to converge to $u_{rg-di}$. By substituting Equation (27) into the following equation, and defining $f\left(´\right)=a_{11i}u_{rgi}+a_{12i}u_{rgi}^{2}+b_{1i}\Omega_{fui}$, we obtain Equation (35).

(34) $${\dot{u}}_{rgi}=a_{11i}u_{rgi}+a_{12i}u_{rgi}^{2}+b_{1i}\tau_{ui}+b_{1i}\Omega_{fui}$$

(35) $${\dot{u}}_{rgi}=f\left(´\right)+b_{1i}\frac{f_{transi}+\beta_{u1i}\left(u_{rg-di}-u_{rgi}\right)-u_{obs2i}}{b_{u0i}}$$

where $b_{1i}\approx b_{ui}$, $f\left(´\right)\approx {\hat{u}}_{2i}$, $f_{transi}\approx {\dot{u}}_{rg-di}$. Then, Equation (35) is written as follows:

(36) $${\dot{u}}_{rgi}-{\dot{u}}_{rg-di}=\beta_{ui}\left(u_{rgi}-u_{rg-di}\right)$$

Defining ${\tilde{u}}_{rgi}=u_{rgi}-u_{rg-di}$, we can obtain:

(37) $${\dot{\tilde{u}}}_{rgi}=-\beta_{ui}{\tilde{u}}_{rgi}$$

where $\beta_{ui}>0$. Finally, it is easily obtained that ${\tilde{u}}_{rgi}\to 0,t\to \infty$. It also means that $\tau_{ui}$ is able to drive $u_{rgi}$ to converge to $u_{rg-di}$. □

As demonstrated above, the controller developed in this paper can effectively drive the robots to fulfill the formation tracking mission.

## 4. Simulation Results and Analysis

In order to verify the effectiveness of the proposed formation control algorithm, a series of simulations were conducted.

### 4.1. Parameters Setting for Simulations

The simulation system consisted of three robots which formed an equilateral triangular formation with a side length of $2\sqrt{3}$ m. The parameters of the above formation controllers for the three robots were set to be identical in the simulation, where $k_{1u}=k_{1\nu }=k_{1w}=2$. The Laplacian matrix was set as follows:

(38) $$L_{4}=\left[\begin{array}{cccc} 1 & 0 & -1 & -1\\ -1 & 1 & 0 & -1\\ 0 & -1 & 1 & -1\\ 0 & 0 & 0 & 0 \end{array}\right]$$

**Figure 9:**
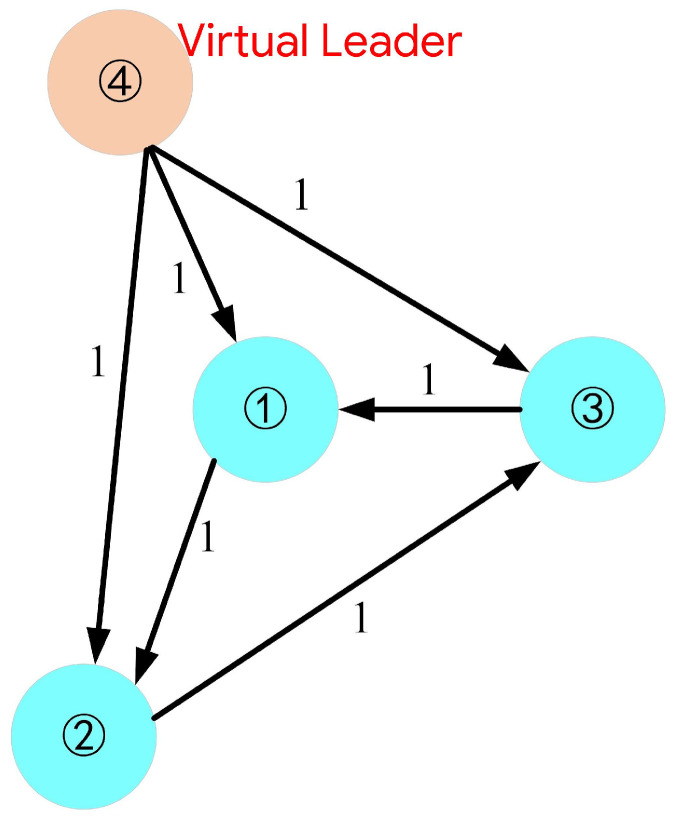
Communication topology of the formation composed of three robots.

The communication topology is depicted in Figure 9. The numbers in the figure refer to the IDs of robots in the multi-robot system. Without loss of generality, three-dimensional straight-line and three-dimensional spiral trajectories were selected as the desired formation trajectories. For comparison with the method proposed in this paper, another algorithm denoted as VS-POP-PID was used in the simulation. The VS-POP-PID algorithm computes the desired positions of each robot according to the formation trajectory and then achieves position tracking based on a parallel proportional–integral–derivative (PID) controller.

### 4.2. Simulation Result Analysis of Three-Dimensional Straight-Line Trajectory Tracking

The equation of the desired formation tracking trajectory is described as follows:

(39) xref=0.1t,0≤t≤60yref=0.1t,0≤t≤60zref=−0.05t,0≤t≤60uref=0.1 νref=0.1 wref=−0.05 

In practical underwater operations, the BUSRs are mainly affected by low-frequency, slowly varying ocean currents caused by water flow oscillation. To better simulate realistic underwater environments, sinusoidal disturbances with different amplitudes and frequencies were selected to imitate these typical hydrodynamic disturbances [23]. Considering that different robots are subject to different water flow disturbance forces, the disturbances acting on the three robots are given as follows:

(40) $$\left\{\begin{array}{c} \Omega_{fu1}=3\sin\left(\frac{\pi }{6}t\right)\\ \Omega_{f\nu 1}=2\sin\left(\frac{\pi }{3}t\right)\\ \Omega_{fw1}=\sin\left(\frac{\pi }{2}t\right) \end{array}\right.$$

(41) $$\left\{\begin{array}{c} \Omega_{fu2}=2\sin\left(\frac{\pi }{3}t\right)\\ \Omega_{f\nu 2}=\sin\left(\frac{\pi }{2}t\right)\\ \Omega_{fw2}=3\sin\left(\frac{\pi }{6}t\right) \end{array}\right.$$

(42) $$\left\{\begin{array}{c} \Omega_{fu3}=\sin\left(\frac{\pi }{2}t\right)\\ \Omega_{f\nu 3}=3\sin\left(\frac{\pi }{6}t\right)\\ \Omega_{fw3}=2\sin\left(\frac{\pi }{3}t\right) \end{array}\right.$$

The above three equations correspond to the disturbances acting on the first, second, and third robot, respectively. The initial positions of the first, second, and third robot were (1 m, 0 m, 1 m), (−3.5 m, 3.5 m, 0 m), (3.5 m, −3.5 m, −1 m), respectively. The sampling time was set to 0.02s. The parameters of the POV-ADRC were set as follows: $\vartheta_{ui}=\vartheta_{\nu i}=\vartheta_{wi}=10$; $\omega_{ui}=\omega_{\nu i}=\omega_{wi}=30$; $b_{ui}=b_{\nu i}=b_{wi}=2.37$; $\beta_{ui}=\beta_{\nu i}=\beta_{wi}=10$(i=1,2,3).

**Figure 10:**
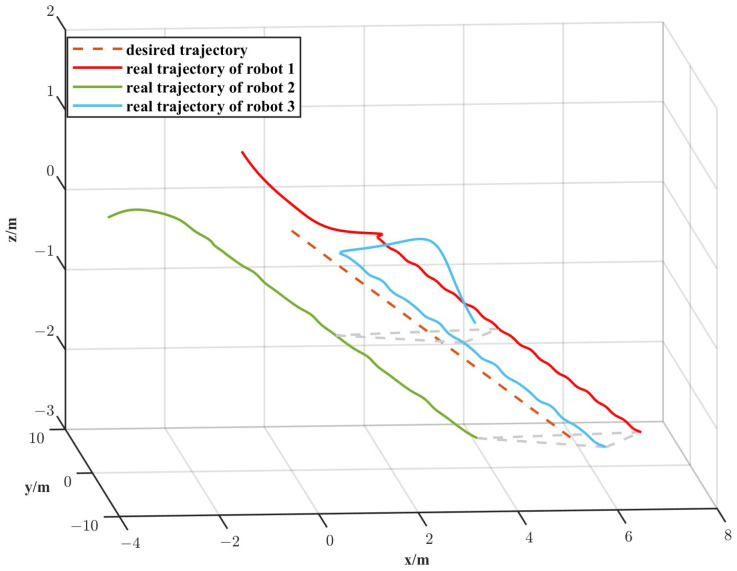
Formation tracking result (3D perspective) of the straight-line trajectory case.

**Figure 11:**
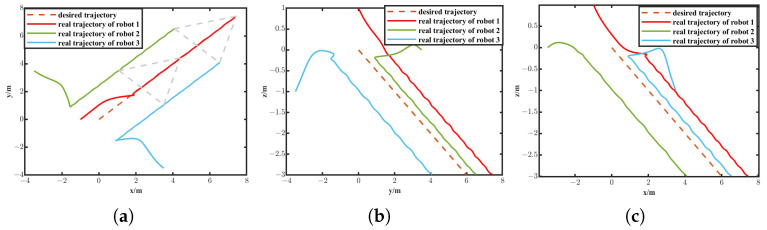
Formation tracking result (2D perspective) of the straight-line trajectory case. (**a**) $X_{I}OY_{I}$. (**b**) $Y_{I}OZ_{I}$. (**c**) $X_{I}OZ_{I}$.

**Figure 12:**
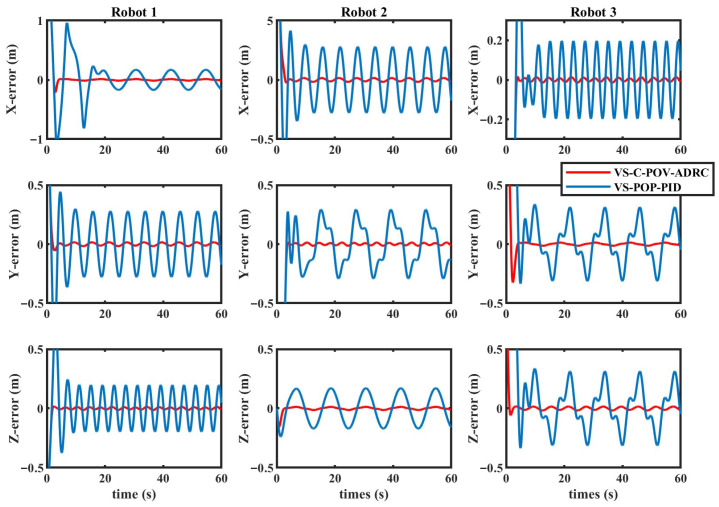
Tracking errors of VS-POP-PID and VS-C-POV-ADRC in the case of straight-line trajectory.

**Figure 13:**
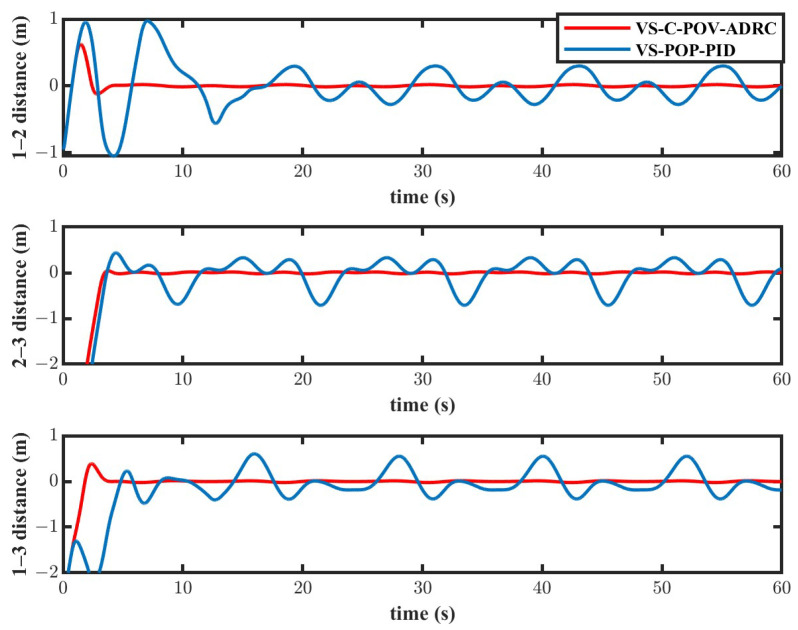
Formation errors of VS-POP-PID and VS-C-POV-ADRC.

**Figure 14:**
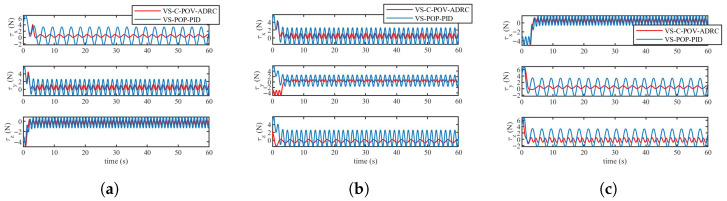
Control inputs of three robots based on two different controllers in the case of straight-line trajectory. (**a**) Input of robot 1. (**b**) Input of robot 2. (**c**) Input of robot 3.

The simulation results are depicted in Figure 10, Figure 11, Figure 12 and Figure 13. The formation trajectory tracking results based on the 3D perspective and the 2D perspective are described in Figure 10 and Figure 11, respectively. The gray dashed line indicates the triangular formation formed by the robots. It can be seen that the three robots are able to form the equilateral triangle and track the desired trajectory. The comparison of tracking errors for each robot in all degrees of freedom along respective trajectories based on the VS-POP-PID and VS-C-POV-ADRC formation controllers are shown in Figure 12. It can be observed that, in comparison with VS-POP-PID, the VS-C-POV-ADRC controller ensures that all robots eliminate obvious environment disturbance and exhibit no obvious oscillations when tracking the corresponding reference trajectories. However, there exists a large oscillation amplitude based on the VS-POP-PID controller. The specific mean square trajectory tracking errors for each robot along all degrees of freedom based on the VS-C-POV-ADRC controller are presented in Table 3. According to Figure 12, each robot tracks the desired trajectory after 5 s. Therefore, to better reflect the steady-state errors after convergence, error calculations commenced at 5 s once the multi-robot formation had stabilized. The error of the desired and real distance between each two robots is labeled as formation error in Figure 13. The formation error based on VS-C-POV-ADRC converges to zero after 5 s, which indicates that the formation maintains a regular triangular shape throughout the entire 3D straight-line trajectory tracking task. In contrast, the formation error based on VS-POP-PID fluctuates continuously, and the maximum error even reaches 0.5 m, which illustrates that the three robots are not able to form the desired formation shape. The comparisons of control inputs based on the two control methods are shown in Figure 14. It can be found that the proposed VS-C-POV-ADRC controller generates smoother, smaller-amplitude control inputs.

### 4.3. Simulation Result Analysis of Three-Dimensional Spiral Trajectory Tracking

The equation of the desired formation tracking trajectory was as follows:

(43) xref=2cos(π15t) yref=2sin(π15t) zref=−0.05t uref=2π15(−sinπ15t) νref=2π15cosπ15t wref=−0.05,0≤t≤60

The disturbances acting on the first robot, second and third robot were as follows:

(44) $$\left\{\begin{array}{c} \Omega_{fu1}=3\sin\left(\frac{2\pi }{3}t\right)\\ \Omega_{f\nu 1}=2\sin\left(\frac{4\pi }{3}t\right)\\ \Omega_{fw1}=\sin\left(2\pi t\right) \end{array}\right.$$

(45) $$\left\{\begin{array}{c} \Omega_{fu2}=2\sin\left(\frac{4\pi }{3}t\right)\\ \Omega_{f\nu 2}=\sin\left(2\pi t\right)\\ \Omega_{fw2}=3\sin\left(\frac{2\pi }{3}t\right) \end{array}\right.$$

(46) $$\left\{\begin{array}{c} \Omega_{fu3}=\sin\left(2\pi t\right)\\ \Omega_{f\nu 3}=3\sin\left(\frac{2\pi }{3}t\right)\\ \Omega_{fw3}=2\sin\left(\frac{4\pi }{3}t\right) \end{array}\right.$$

**Figure 15:**
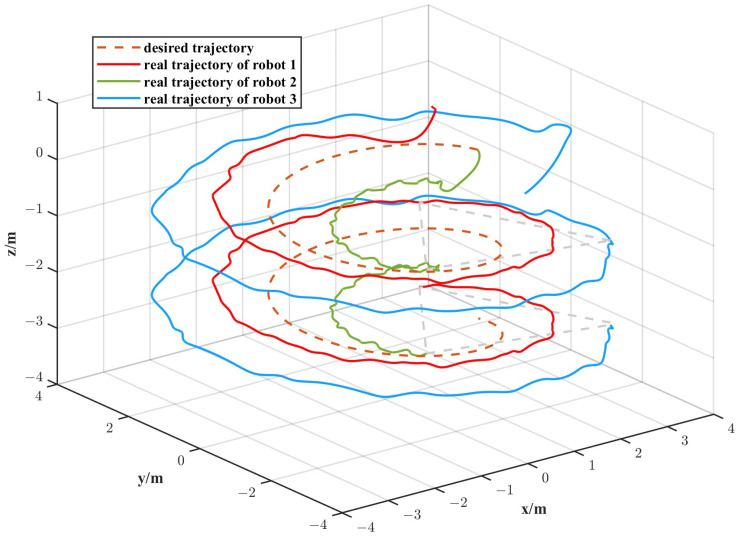
Formation tracking result (3D perspective) of the spiral trajectory case.

**Figure 16:**
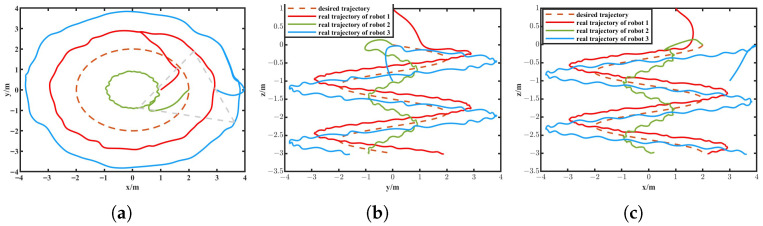
Formation tracking result (2D perspective) of the spiral trajectory case. (**a**) $X_{I}OY_{I}$. (**b**) $Y_{I}OZ_{I}$. (**c**) $X_{I}OZ_{I}$.

**Figure 17:**
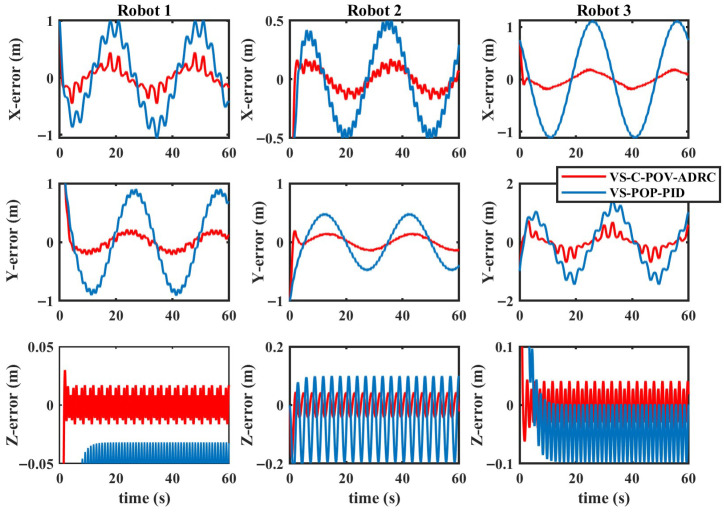
Tracking errors of VS-POP-PID and VS-C-POV-ADRC in the case of spiral trajectory.

**Figure 18:**
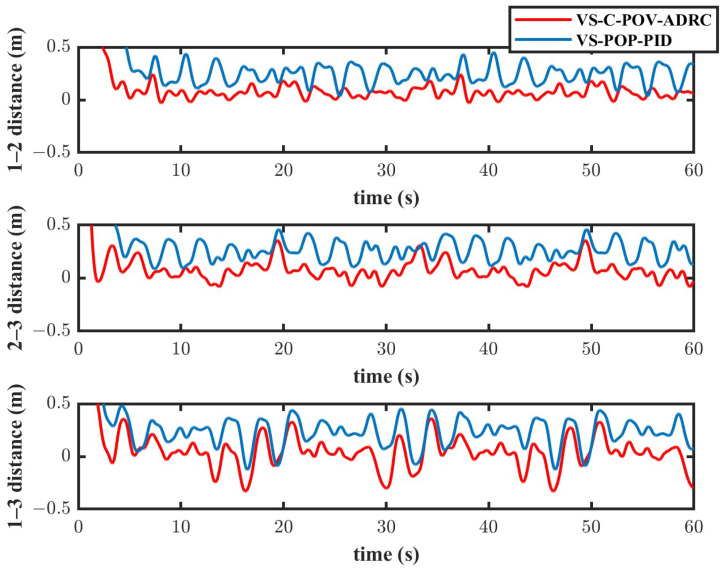
Distance errors of VS-POP-PID and VS-C-POV-ADRC.

**Figure 19:**
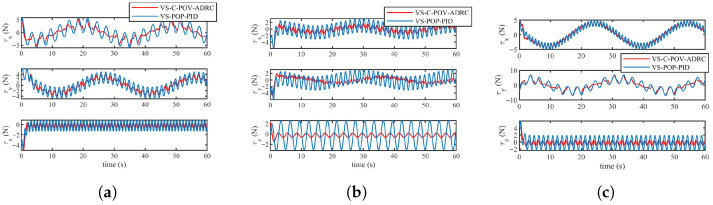
Control inputs of three robots based on two different controllers in the case of spiral trajectory. (**a**) Input of robot 1. (**b**) Input of robot 2. (**c**) Input of robot 3.

The initial positions of the first, second and third robot were (1 m, 0 m, 1 m), (2 m, 0 m, 0 m), (3 m, 0 m, −1 m), respectively. The sampling time and the parameters of the VS-C-POV-ADRC were all consistent with those in Section 4.2. The formation tracking results are shown in Figure 15 and Figure 16. The gray dash line indicates the triangular formation. The legend of Figure 16a is consist with that of Figure 16b. Each robot was able to track the corresponding desired trajectory and form the desired formation shape. The tracking errors based on VS-C-POV-ADRC and VS-POP-PID are compared in Figure 17. It can be seen that in contrast to VS-POP-PID, the tracking errors based on the VS-C-POV-ADRC controller almost converge to zero in three directions and exhibit no obvious oscillations under strong disturbance. However, the tracking error based on the VS-POP-PID method not only exhibits a large oscillation amplitude but also has a non-zero median during that oscillation, which indicates a significant steady-state error. The concrete tracking errors based on VS-C-POV-ADRC are listed in Table 4. The formation errors between each two robots are described in Figure 18. The formation errors based on the VS-C-POV-ADRC almost converge to zero, and there exist slightly minor oscillations, which illustrates that the formation basically maintains a regular triangular configuration while tracking the 3D spiral trajectory. The comparisons of control inputs based on the two control methods are shown in Figure 19. The above simulation results demonstrate that the controller proposed in this paper performs well even under spiral formation trajectories and strong environmental disturbance.

Overall, the VS-C-POV-ADRC formation tracking controller proposed in this paper is able to drive robots to accomplish trajectory tracking tasks while maintaining the formation shape stably under strong disturbance.

## 5. Experiment Results and Analysis

**Figure 20:**
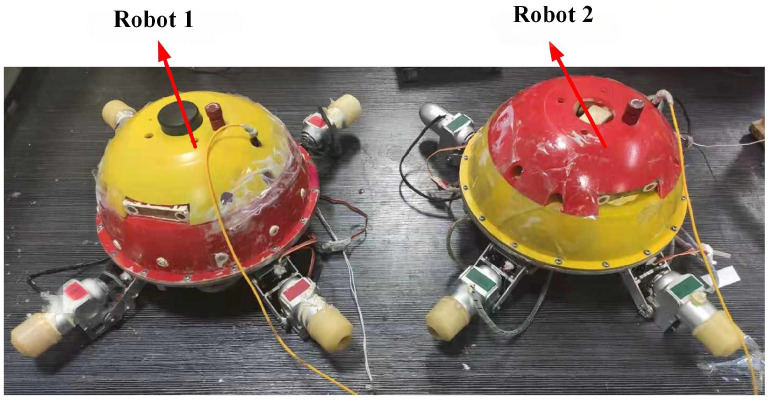
The two spherical robots used in the experiment.

In this section, an experiment was carried out to further validate the performance of the algorithm proposed in this paper. To this end, a real robot formation system composed of two BUSRs was developed as shown in Figure 20.

### 5.1. Experiment Setting

**Figure 21:**
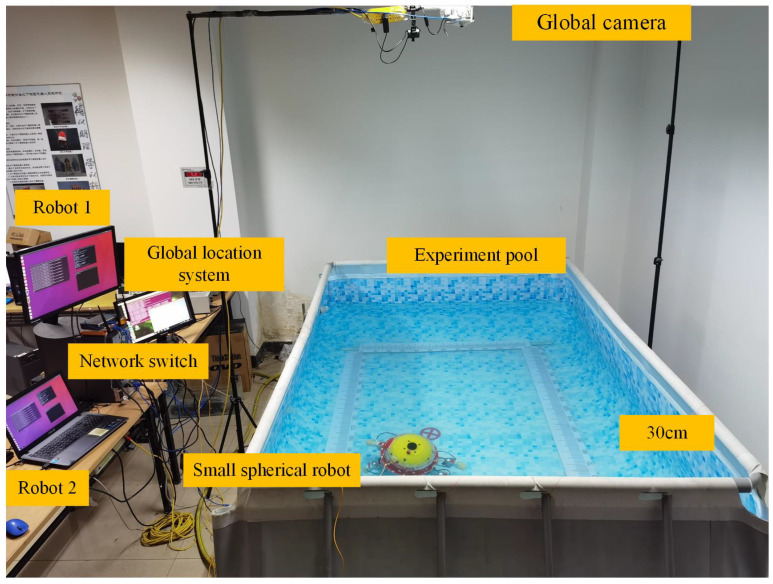
Experiment environment.

**Figure 22:**
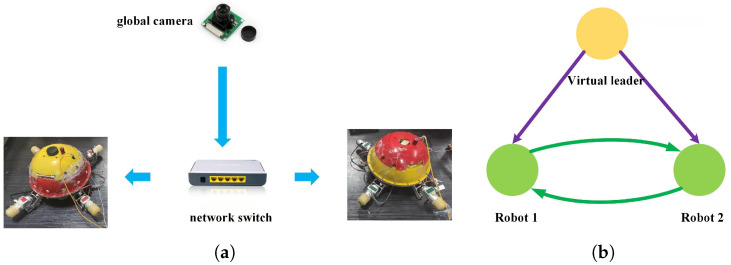
Information interaction for the formation system. (**a**) Data transmitting. (**b**) Communication topology.

The experiment was carried out in an indoor pool with a dimension of 3.5 m × 2.5 m × 1 m (length × width × height), as shown in Figure 21. The framework of the multi-spherical robot formation system mainly consisted of a global positioning system based on a global camera, two BUSRs, and a wireless AP/router. The communication relationship is depicted in Figure 22a. The global camera performed real-time position detection for the two robots. In order to ensure position acquisition, the two robots floated on the water surface. The positions of the two robots were transmitted via an industrial switch over optical fibers, which guaranteed fully real-time data transmission. In this way, each robot received not only its own position information but also that of the other one. Figure 22b describes the communication topology of the formation system. The global camera acted as a virtual leader, which sent the formation reference trajectory to the two robots. Based on the trajectory, the two robots reproduced the corresponding reference trajectories based on the regular polygonal virtual structure method. In the experiment, the two robots were required to form a straight-line formation shape and track a rectangular trajectory. The equation of the desired rectangular trajectory was as follows:

(47) xref=0.45−16Trealt yref=0.45 uref=−16Treal νref=0,0≤t≤Treal4

(48) xref=−0.45 yref=0.45−16Trealt uref=0 νref=−16Treal,Treal4≤t≤Treal2

(49) xref=−0.45+16Trealt yref=−0.45 uref=16Treal νref=0,Treal2≤t≤3Treal4

(50) xref=0.45 yref=−0.45+16Trealt uref=0 νref=16Treal,3Treal4≤t≤Treal

The center position of the desired rectangular trajectory was (0,0), and the side length was 0.9 m. The sampling time was consistent with that in the previous simulation. The running time $T_{real}$ was 120 s. Parameters of the VS-C-POV-ADRC algorithm were set as follows: $a_{12}=a_{21}=0.25$, $k_{1u}=k_{1\nu }=0.2$, $\beta_{ui}=\beta_{\nu i}=0.8$, $\vartheta_{ui}=\vartheta_{\nu i}=5$, $\omega_{ui}=\omega_{\nu i}=10$, $b_{ui}=b_{\nu i}=1$(i=1,2). The initial positions of robot 1 and robot 2 were (0.85 m, 0.97 m) and (−0.34 m, 1.02 m), respectively.

### 5.2. Experiment Results

**Figure 23:**
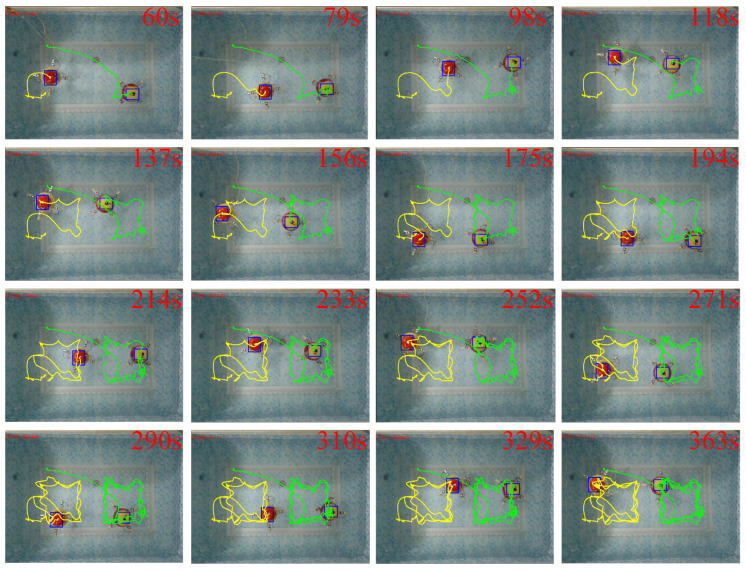
Process of the formation tracking.

**Figure 24:**
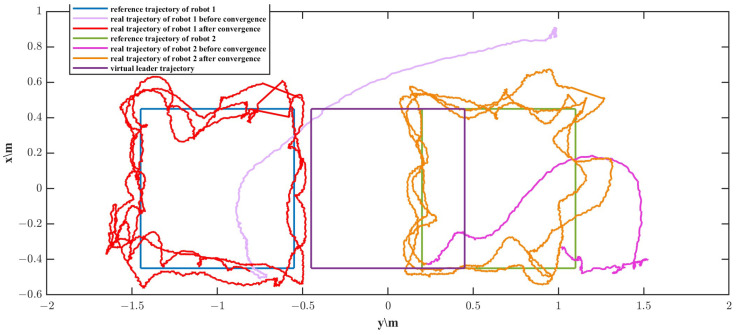
Contrast between desired formation trajectory and real trajectory.

In order to observe the experiment results, the two robots tracked the desired trajectory three times. A series of temporal visualization during the process of the rectangular trajectory formation tracking is depicted in Figure 23. The green line and yellow line represent the movement trajectory of robot 1 and robot 2, respectively. Since the initial position of robot 1 deviates significantly from the reference trajectory, the robot conducts a rapid movement in the early stage. A comparison between the reference and actual trajectories of the two robots is presented in Figure 24. It can be seen that during the formation tracking, the process is divided into two phases. The first phase is to form the desired formation shape, and the second phase is to track the reference trajectory and to maintain the formation.

**Figure 25:**
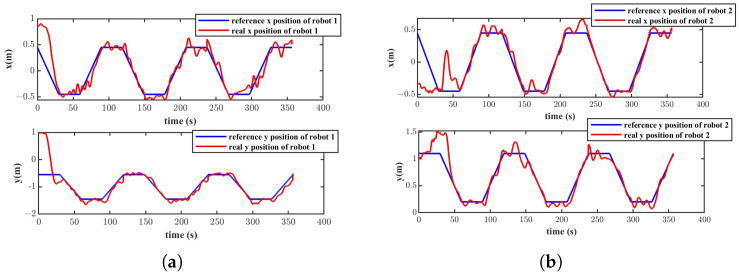
Tracking trajectory of the two robots in X and Y directions (**a**) Tracking trajectory of robot 1. (**b**) Tracking trajectory of robot 2.

**Figure 26:**
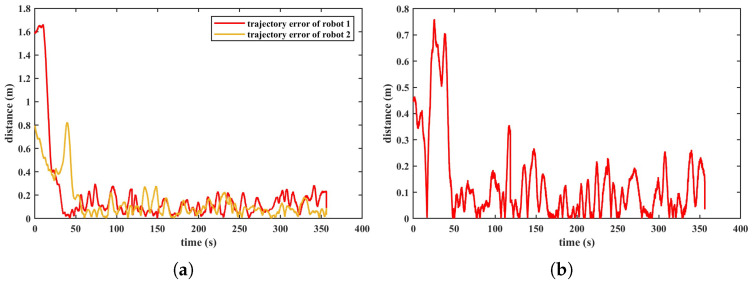
Error of trajectory tracking and formation (**a**) Error of trajectory tracking. (**b**) Error of formation.

The real and desired positions of the two robots in the X and Y directions are depicted in Figure 25. As shown in the figure, the two robots form the straight-line formation shape and track the corresponding trajectory after 70 s. Due to the small size of the experimental water tank, the reflection of water flow off the tank walls, and the water flow disturbance generated by thrusters lead to a complex flow field in the experiment tank. Therefore, slight oscillations were exhibited when the two robots tracked the desired trajectories. Figure 26a presents the Euclidean distance errors between robot 1, robot 2 and their respective reference trajectories. The trajectory tracking errors of robot 1 and robot 2 exhibit large transient peaks in the initial phase (0–50 s). The errors then converge rapidly and enter a steady state. Figure 26b presents the absolute values of the distance errors that refer to errors between their reference and actual relative distance. The distance error also presents a transient peak of around 0.75 m at the beginning, followed by a quick decay, and then it enters a steady state. The Euclidean trajectory errors of the two robots and the formation distance error after convergence are shown in Table 5. As described in the above table, the median tracking errors of robot 1 and robot 2 are 8.29 cm and 4.12 cm respectively. The median formation error is 6.99 cm. According to Figure 26 and Table 5, the trajectory tracking errors are less than the body length and the formation error is approximately 18% of the formation desired distance, which illustrates that the VS-C-POV-ADRC is able to control the multiple robots to achieve formation and trajectory tracking tasks.

## 6. Discussion

On the basis of the simulation and experiment analysis in Section 4 and Section 5, the proposed formation tracking method was demonstrated to be feasible and effective for multiple BUSRs. In particular, the flow disturbance of the small pool was highly complex in the experiment, and each robot was subject to extremely severe external disturbances. Nevertheless, even under such intense external disturbances, the VS-C-POV-ADRC algorithm could still achieve formation trajectory tracking with an accuracy of approximately one body length. The excellent tracking accuracy and strong robustness mainly stem from two key designs of the proposed method. First, the dual-loop consensus-ADRC framework ensures formation coordination through the outer consensus loop, and uses the inner POV-ADRC to actively estimate and compensate for unmodeled dynamics and external environmental disturbances in real time. Second, the rotating global (RG) coordinate system eliminates trigonometric nonlinear terms and stabilizes the bandwidth of the extended state observer, which significantly improves the precision of disturbance observation and tracking stability.

The formation method proposed in this paper was designed under a fixed and connected communication graph with a virtual leader structure. All robots could receive information from the virtual leader in real time, which ensured reliable consensus convergence and stable formation control even under strong current disturbances. However, the proposed approach has several known limitations. First, communication delays were not considered in the current design. Excessive or time-varying communication delays will reduce the convergence speed, increase tracking errors, and even affect the stability of the formation system. Second, if the communication topology switches frequently or becomes disconnected, the consensus convergence may be slowed down or interrupted, and formation accuracy will degrade.

To this end, a distributed consensus algorithm with event-triggered or self-triggered mechanisms will be combined with ADRC in future work to enhance the adaptability to switching communication topologies and expand the application to large-scale swarm systems.

## 7. Conclusions

In this paper, the formation tracking problem for multiple biomimetic underwater spherical robots was studied. This study aimed to find a method with a simple structure, a small number of tunable parameters, convenient parameter tuning and anti-disturbance capability to promote the engineering implementation and practical application. To this end, a VS-C-POV-ADRC formation tracking controller integrating a virtual structure (VS), consensus protocol and parallel output-velocity-type active disturbance rejection control (POV-ADRC) was proposed. Firstly, the reference positions of each BUSR were obtained according to the global reference trajectory and defined formation shape based on the virtual-structure strategy. Then, a rotating global (RG) coordinate system was designed to decouple the position and heading angle of BUSRs, which eliminated the trigonometric nonlinear terms in the linear extended state observer (LESO) of ADRC and simplified the parameter tuning process. Further, a double-loop control architecture was constructed for the multi-BUSR system. The outer consensus control loop generated the desired velocity for each robot based on the reference position to ensure formation consistency. The inner POV-ADRC loop achieved high-precision velocity tracking with strong anti-disturbance performance. The proposed controller had only five adjustable parameters per motion direction to fit the limited computing capacity of BUSRs. The theoretical stability was proved under the condition of reasonable parameter settings. Finally, simulations of three BUSRs forming an equilateral triangular formation to track 3D straight-line and spiral trajectories under sinusoidal water flow disturbances were carried out. The simulation results showed that the VS-C-POV-ADRC method maintained a stable formation, and formation error converged to zero within 5 s, significantly outperforming the VS-POP-PID comparison method in anti-disturbance and tracking stability. Physical experiments of two BUSRs tracking a rectangular trajectory in an indoor pool with complex flow field disturbances were designed. The experiment results showed that the tracking and formation errors of the system were all within 30 cm (one robot body length), which proved the effectiveness of the proposed formation tracking method.

This research provides a feasible and practical engineering solution for the formation tracking control of multiple BUSRs. However, the proposed method is based on the centralized control assumption that all robots can receive the information of the virtual leader in real time, which limits its application in large-scale swarm formation. In the future, a distributed consensus algorithm with event-triggered or self-triggered mechanisms will be combined with ADRC to enhance the adaptability to switching communication topologies and expand the application to large-scale swarm systems.

## Figures and Tables

**Table 1:** Technical parameters of the robot.

Items	Parameters
Dimension (width × length × height)	30 cm × 60 cm × 30 cm
Mass in air	6.5 kg
Max thrust	3.8 N
Propulsion mode	X-shaped four-thruster vector propulsion system
Max velocity	0.5 m/s
Embedded computing platform	NVIDIA Jetson TK1
Micro controller unit	STM32F407ZGT6
Sensors	Pressure sensor (MS5803-14BA) IMU (3DM-GX5-45) Stereo camera Acoustic communication module (Micron Sonar)
Power	7.4 V rechargeable Ni-MH batteries (13,200 mAh)
Operation time	Average 100 min

**Table 2:** Hydrodynamic parameters of the BUSR.

Parameter	Value	Parameter	Value	Parameter	Value
$a_{11}$	−11.4387	$a_{12}$	0.0196	$b_{1}$	2.3742
$a_{21}$	−11.4506	$a_{22}$	0.0199	$b_{2}$	2.3742
$a_{31}$	−11.5714	$a_{32}$	0.0204	$b_{3}$	2.4272
$a_{41}$	−0.583	$a_{42}$	0.001557	$b_{4}$	4.7619

**Table 3:** Mean square errors of 3D straight-line trajectory tracking based on VS-C-POV-ADRC.

-	Robot 1	Robot 2	Robot 3
Along $X_{I}$ direction (m)	0.0009	0.0006	0.0001
Along $Y_{I}$ direction (m)	0.0006	0.0001	0.0009
Along $Z_{I}$ direction (m)	0.0001	0.0009	0.0006

**Table 4:** Mean square error of 3D spiral trajectory tracking based on VS-C-POV-ADRC.

-	Robot 1	Robot 2	Robot 3
along $X_{I}$ direction (m)	0.0353	0.0089	0.0140
along $Y_{I}$ direction (m)	0.0155	0.0092	0.0735
along $Z_{I}$ direction (m)	0.0001	0.0009	0.0006

**Table 5:** Tracking and formation errors.

-	Min	Max	Median
Robot 1 Euclidean trajectory errors (m)	0.0003	0.3012	0.0829
Robot 2 Euclidean trajectory errors (m)	0.0001	0.2253	0.0412
Formation distance error (m)	0.0009	0.3560	0.0699

## Data Availability

Data is contained within the article.
